# Serum complement factor I is associated with disease activity of systemic lupus erythematosus

**DOI:** 10.18632/oncotarget.23907

**Published:** 2018-01-03

**Authors:** Min-Hua Tseng, Shih-Hua Lin, Chao-Yi Wu, Hui-Ping Chien, Huang-Yu Yang, Yung-Chang Chen, Yu-Ching Chou, Jing-Long Huang

**Affiliations:** ^1^ Division of Nephrology, Department of Pediatrics, Chang Gung Memorial Hospital and Chang Gung University, Taoyuan, Taiwan; ^2^ Division of Nephrology, Department of Medicine, Tri-Service General Hospital, Taipei, Taiwan; ^3^ Division of Allergy, Asthma and Rheumatology, Department of Pediatrics, Chang Gung Memorial Hospital and Chang Gung University, Taoyuan, Taiwan; ^4^ Department of Pathology, Tri-Service General Hospital, Taipei, Taiwan; ^5^ Department of Nephrology, Chang Gung Memorial Hospital and Chang Gung University, Taoyuan, Taiwan; ^6^ School of Public Health, National Defense Medical Center, Taipei, Taiwan

**Keywords:** systemic lupus erythematosus, disease activity, complement regulatory proteins, lupus nephritis, biomarker

## Abstract

Although aberrant complement activation is involved in the pathogenesis of systemic lupus erythematosus (SLE), the role of complement regulatory proteins in disease activity of SLE remains limited. We enrolled the pediatric-onset SLE patients from our cohort study over 10 years. The clinical and laboratory data including SLEDAI disease activity score, and serum complement factor H (CFH), CFI, CD46, C5a, and C5b-9 in the active and remission phases were determined. Glomerular C5b-9 deposition as a complement activity marker was also examined. Forty patients (35 female and 5 male, aged 13.9 ± 3.8 years) met the criteria of investigation were assessed. Fever and kidney were the most common symptom and organ involved, respectively. Mean SLEDAI in the active and remission phases were 12.6 vs 1.7, respectively. All patients exhibited lower serum C3, C4, CFH and CFI and higher serum anti-dsDNA and CD46 in the active pahse. There was a significant difference in serum CFH, CFI and CD46 between active and remissive phases. Serum CFI but not CFH and CD46 level was negatively correlated with SLEDAI score in active phase. Compared to classical activity markers, serum CFI was superior to C4 and anti-dsDNA in reflecting disease activity and also significantly correlated with white blood count and hemoglobin. Glomerular C5b-9 depositions were detected in patients with nephritis during active phase but not in disease controls. Serum CFI level may not only be a promising biomarker for disease activity of SLE, but also reflects the hematological features of SLE.

## INTRODUCTION

The aberrant complement system resulting in loss of self-tolerance, is one of the abnormalities of the immune system involved in the etiopathogenesis of systemic lupus erythematosus (SLE). Complement deficiency, including C1, C2 and C4, predisposed the development of SLE by impairment of physiological waste disposal mechanisms [1, 2]. Paradoxically, the excessive generation of end complement products caused by complement activation at the site of immune complex deposition leads to tissue inflammation and injury [3, 4]. Complement activation is a proteolytic cascade tightly controlled by complement regulatory proteins (CRPs) including cell membrane-bound (CD46) and soluble proteins (Complement factor H/CFH and complement factor I/CFI). Deficiency or defects in CFH and CFI are associated with complement-mediated hemolytic uremic syndrome, hemolysis, and thrombosis. To date, the activity of soluble and membrane-bound CRPs responsible for modulating the severity of disease in patients with SLE remains unexplored.

Despite the advance in the medical care of SLE in the past decades, the outcome of SLE remains ungratified. The major challenge of this is the activity monitoring of patients with SLE. Although the complement C3, C4, and anti-dsDNA are conventionally considered as biomarkers for the disease flares of SLE, recent studies have provided some caveats with these laboratory markers of disease activity [5–7]. The unpredictable clinical course and the lack of reliable markers for SLE hamper timely recognition and appropriate treatment. Until now, study for evaluating the CRPs as predictors of lupus activity seems limited.

Currently, we have collected a larger number of pediatric-onset SLE patients and aimed to determine the alteration of serum CRPs during flares and their associations with the disease activity of SLE. Results to be reported indicate that serum CFI level may not only be a promising biomarker for disease activity and hematological features of SLE. To the best of our knowledge, this is the first study focusing on the change in serum CFI levels and disease activity of SLE.

## RESULTS

### Demographic and clinical features

**Figure 1:**
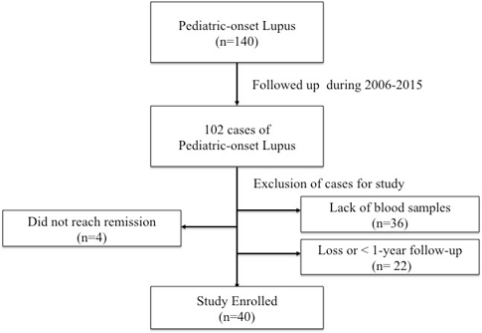
Patients enrollment flow chart

Forty lupus patients (35 female and 5 male, Figure 1) with mean age of 13.9 ± 3.8 years (range, 5.0-17.5 years) and mean follow-up duration of 6.5 ± 4.3 years were enrolled during this study period. As shown in Table 1, fever, malar rash, joint pain or swelling, and neuropsychiatric symptoms were the most common features in the active phase. The kidney (29/40) was the most common organ involvement. Twenty-five patients (25/29) had biopsy-proven nephritis with proliferative lupus nephritis (22/25) being the most dominant.

**Table 1:** Demographic, clinical characteristics and outcome in patients with lupus

	Patients (*n =* 40) (%)
Age/Sex (female/male)	13.9 ± 3.8 (35/5)
Manifestations during the active phase	
Fever	26 (65)
Malar rash and/or discoid rash	18 (45)
Pleuritis and/or pericarditis	11 (27.5)
Oral ulcers	4 (10)
Non-erosive arthritis	16 (40)
Photosensitivity	11 (27.5)
Hematologic disorders	17 (42.5)
Neurologic disorders	15 (37.5)
Renal disorders/Histologically proven nephritis	29 (72.5) / 25 (62.5)
Proliferative nephritis	22 (55)
Non-proliferative nephritis	3 (7.5)
Vasculitis	8 (20)
Treatment	
Corticosteroid	40 (100)
Hydrochloroquine	9 (22.5)
Mycophenolate mefetil	3 (7.5)
Intravenous cyclophosphamide	19 (47.5)
Azathioprine	15 (37.5)
Clinical outcome	
Sequelae	3 (7.5)

### Biochemical characteristics

The mean SLEDAI values in the active and remission phases were 12.57 versus 1.70, respectively. The mean renal SLEDAI values were 4.25 and 0.45 in the active and remission phase, respectively. Patients with nephritis had higher mean SLEDAI score than those without nephritis (12.00 versus 9.73, *p* < 0.05). Patients in the active phase had significantly lower C3, C4, hemoglobin, and platelet levels and higher serum anti-ds DNA and creatinine levels than those in the remission phase. Compared with the remission phase, patients in the active phase had higher proportions of proteinuria and hematuria (Table 2).

**Table 2:** Disease activity and laboratory data in the active and remission phases

	Active	Remission	*p* value
SLEDAI	12.57 (10.32–15.81)	1.70 (0.78– 2.65)	< 0.01
Renal SLEDAI	4.25 (3.02–5.47)	0.45 (0.09–0.80)	< 0.001
White blood cell (/μL)	4,460 (3,491–5,429)	5,392 (4,511–6,273)	0.101
Hemoglobin (gm/dL)	10.5 (9.7–11.2)	12.5 (11.9–13.0)	< 0.001
Platelet (1000/μL)	180.3(146.3–214.2)	239.1 (215.3–262.8)	0.001
Creatinine (mg/dL)	0.76 (0.65–0.87)	0.63 (0.57–0.69)	0.026
eGFR (ml/min/1.73 m2)	84.2 (74.6– 98.8)	102 (94.4–114.2)	0.026
Proteinuria (%)	28/40 (70)	4 (10)	< 0.001
Hematuria (%)	29/40 (72)	6 (15)	< 0.001
C3 (mg/dL)	46.53 (38.86–54.15)	85.56 (78.68–92.45)	< 0.001
C4 (mg/dL)	6.21 (4.95–7.47)	12.54 (10.58–14.50)	< 0.001
Anti–dsDNA (unit/ml)	523.17 (316.14–730.19)	141.00 (80.22–201.77)	< 0.001

### Serum complement regulatory proteins and terminal complement products

**Figure 2:**
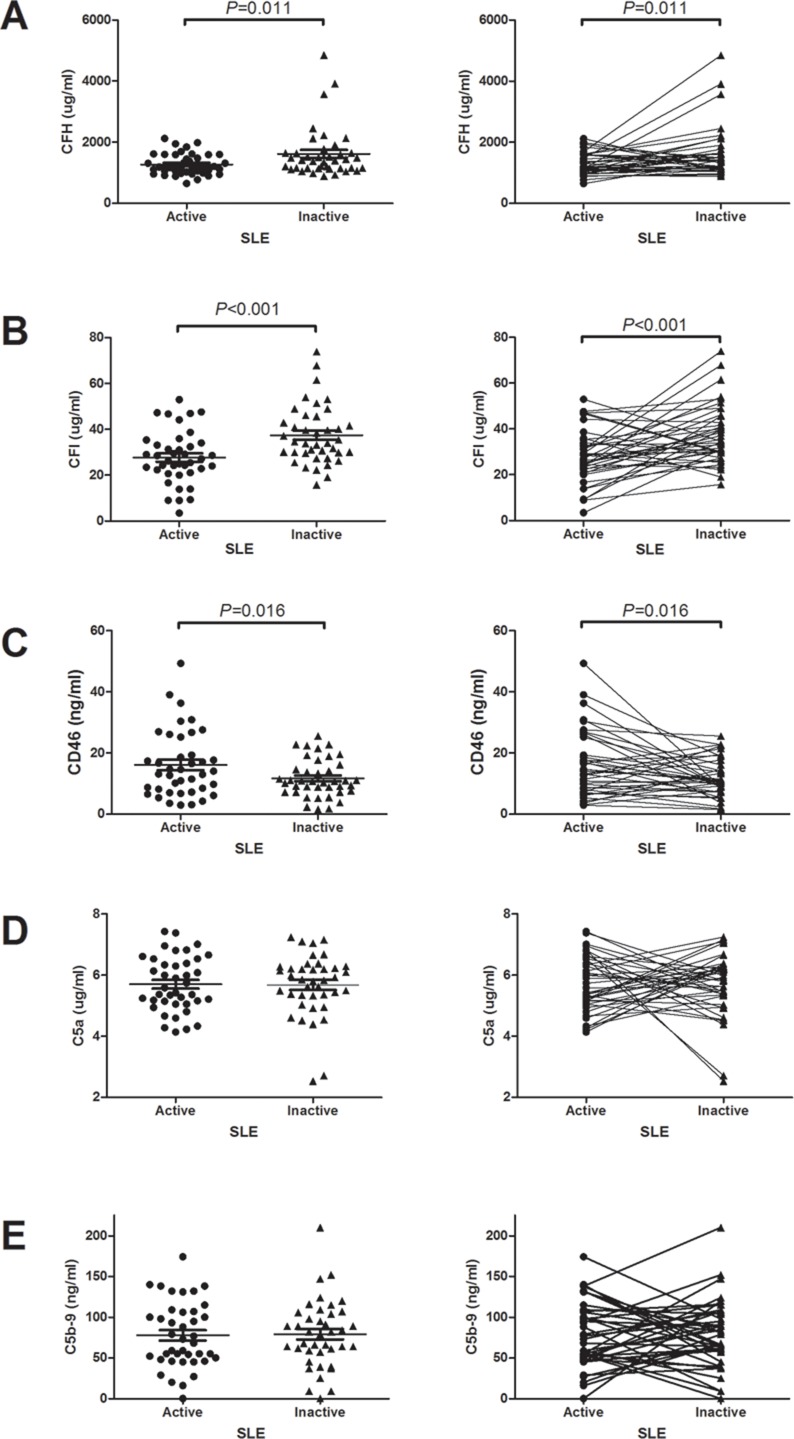
The changes of serum complement regulatory proteins and terminal complement products levels between active and remission phases (**A**) Serum CFH levels. (**B**) Serum CFI levels. (**C**) Serum CD46 levels. (**D**) Serum C5a levels. (**E**) Serum C5b-9 levels.

The mean serum levels of complement factor H, I in the active phase were significantly lower than those in the remission phase (1261.9 ± 349.7 *vs* 1535.4 ± 638.2 μg/ml, *p* = 0.011, 27.7 ± 11.5 *vs* 37.5 ± 12.8 μg/ml, *p* < 0.001, respectively) (Figure 2A and 2B). Compared to remission phase, the mean serum levels of CD46 in the active phase were significantly higher (16.1 ± 10.8 *vs* 11.7 ± 6.2, *p* = 0.016) (Figure 2C). The serum C5a levels in the active and remission phases were 5.7 ± 0.9 and 5.6 ± 1.0 μg/ml, respectively. The serum C5b-9 levels were 77.7 ± 40.3 and 78.9 ± 41.5 μg/ml in the active and remission phases, respectively. There were no significant differences in the serum C5a and C5b-9 levels between the active and remission phases (Figure 2D and 2E). Because patients with and without nephritis may have different characteristics of complement activation, we analyzed accordingly. As shown in Table 3, the results showed that no significant difference between patients with and without nephritis on the serum C3, C4, C5a, C5b-9, CFH, CFI and CD 46 on both active and remission phases.

**Table 3:** Complement characteristics of lupus patients with and without nephritis

	Patients with nephritis	Patients without nephritis	*P* value
Active			
CFH (ug/ml)	1414.22 ± 140.63	1186.31 ± 79.70	0.356
CFI (ug/ml)	28.55 ± 2.18	25.50 ± 3.40	0.575
C3	44.90 ± 4.03	50.74 ± 5.47	0.243
CD46 (ng/ml)	16.61 ± 2.00	14.70 ± 3.39	0.405
C4	6.42 ± 0.56	6.72 ± 1.03	0.791
C5a (ug/ml)	5.82 ± 0.168	5.42 ± 0.20	0.255
C5b–9 (ng/ml)	79.85 ± 8.05	76.72 ± 10.33	0.864
Remission			
CFH (ug/ml)	1549.50 ± 130.24	1516.56 ± 125.13	0.467
CFI (ug/ml)	38.12 ± 2.28	35.62 ± 4.36	0.422
CD46 (ng/ml)	11.54 ± 1.17	12.15 ± 1.85	0.660
C3	85.30 ± 3.5	86.26 ± 5.48	0.976
C4	12.39 ± 0.96	12.96 ± 1.7	0.832
C5a (ug/ml)	5.63 ± 0.19	5.72 ± 0.31	0.950
C5b–9 (ng/ml)	82.45 ± 8.04	69.81 ± 11.00	0.440

### Correlations between serum complement regulatory proteins and disease activity

**Figure 3:**
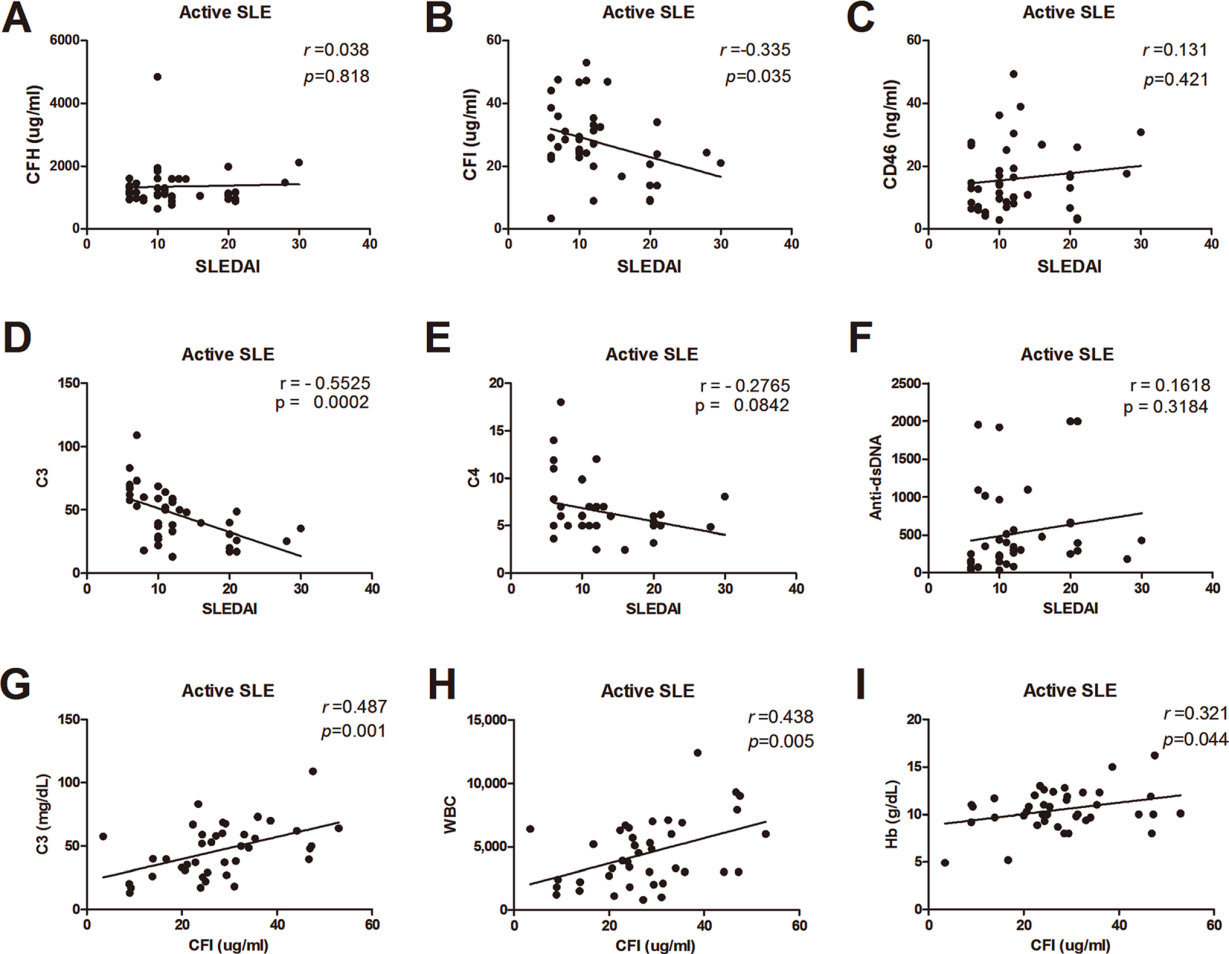
Correlations between serums CRPs, C3, C4, anti-dsDNA, and disease activity (**A**) Serum CFH levels and SLEDAI. (**B**) Serum CFI levels and SLEDAI. (**C**) Serum CD46 levels and SLEDAI. (**D**) Serum C3 levels and SLEDAI. (**E**) Serum C4 levels and SLEDAI. (**F**) Serum anti-dsDNA and SLEDAI. (**G**) Serum CFI levels and C3. (**H**) Serum CFI levels and whit blood count. (**I**) Serum CFI levels and hemoglobin.

Since there were the associations between serum CRPs and disease activity, we analyzed the correlations between SLEDAI and serum CFH, CFI, CD46 and classical activity markers including serum C3, C4, anti-dsDNA to evaluate the usefulness of CRPs as activity biomarkers of SLE. As shown in Figure 3, serum CFI and C3 had correlations with SLEDAI in the active phase. Serum CFI levels were significantly correlated with serum C3, and hematologic laboratory findings such as white blood count, and hemoglobin (Figure 3).

### Immunofluorescence stain of renal tissue

**Figure 4:**
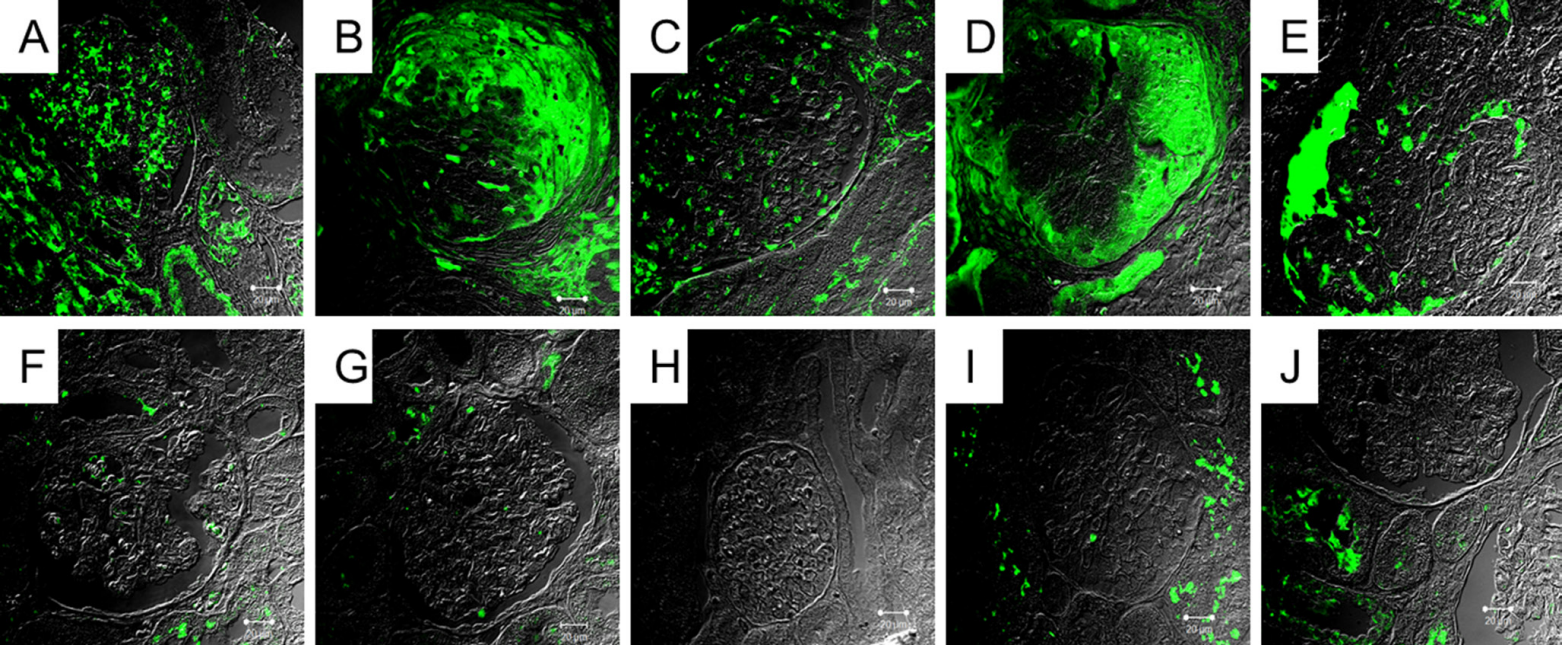
Immunofluorescence stain of C5b-9 on renal tissue from patients with lupus nephritis (**A–E**), normal subjects (**F, G, H**), and disease controls (**I,J**). These represent no stain (G,H,I,J), faint stain (F), intermediate (C), and high stain (A, B , D, E).

Due to no significant change on serum terminal complement products between the active and remission phases, we evaluated the possible presence of tissue deposition of terminal complement components in active phase. The renal tissues obtained from five patients of lupus nephritis in active phase, three normal subjects, and two disease controls with focal segmental glomerulonephritis were studied. As shown in Figure 4, four high and one intermediate depositions of C5b-9 on glomeruli were detected by immunofluorescence stains in all five patients with occurrence of nephritis in active phase, faint stain in one normal subjects, negative stain in other 2 normal subjects and disease controls.

### Treatment and clinical outcome

As shown in Table 1, all patients were treated with corticosteroid treatment during their active phase. Twenty-four (60%) patients received intravenous methylprednisolone pulse therapy due to proliferative nephritis and/or central nervous system lupus. Among 22 patients with biopsy-proven proliferative lupus nephritis, intravenous cyclophosphamide was administered in 19 patients and mycophenolate mofetil was given in another 3 patients. Three patients, 1 intracranial hemorrhage and 2 chronic kidney disease, exhibited sequelae after the flares of disease.

## DISCUSSION

To the best of our knowledge, this is the first study demonstrating the correlation between serum CFI levels and disease activity of SLE. We found that there were significant changes in the serum levels of CFH, CFI and CD46 between the active and remission phase, but only serum CFI levels were statistically correlated with SLEDAI. Serum CFI was not only superior to serum C4 and anti-dsDNA in correlating with SLEDAI score, but also positively correlating with serum C3 and hematological markers including leukocyte count and hemoglobin. In addition, the hypocomplementemia with glomerular depositions of terminal complement products in active phase suggested the exaggerated complement activation. These findings pointed that serum CRPs alter significantly during flares and the serum CFI level is a potential biomarker of SLE activity.

The current study showed significant change of serum CFH, CFI and CD46 levels and the complement activation by demonstrating the findings of the hypocomplementemia in active phase. The explanation of this change could be the following reasons. First, increased consumption of serum CRPs occurs secondary to the process of complement activation. Earlier study which showed that the finding of low serum concentrations of CRPs in patients with active SLE due to increase of catabolism supports this speculation [8]. Second, this change may result from the generation of anti-serum CRPs autoantibodies. Previous reports have demonstrated that the autoantibodies against the complement component C1q, CFH and CFI in patients SLE and complement dysregulation hemolytic uremic syndrome [9–11]. Further studies to identify the circulating anti-CFH, CFI and CD46 autoantibodies and the catabolic production of C3b such as C3c and C3d are needed to clarify the mechanism of alternation of serum CRPs during disease flares.

Our study showed that both CFH and CD46 levels in active phase changed significantly compared to those in remission phase, corroborating earlier observations [12, 13, 14]. In contrary to pervious study which analyzed the correlation of serum CFH and disease activity of patients with lupus nephritis, our study demonstrated that only serum CFI but not CFH and CD46 correlated statistically with SLEDAI scores [13, 15]. The reason of this discrepancy needs to be elucidated, however, the focusing on different consequences in SLE subjects might account for this discrepancy. In this current study, these enrolled patients received regular follow-up evaluations from a long-term cohort study. We believed that the serum CFI truly reflects the disease activity because the serum CFI is strongly correlated with the serum C3 and the hematological markers including leukocyte and platelet counts during disease flares.

Although the elevated serum hallmarks of complement activation, C5b-9 and C5a, have been demonstrated during SLE flares, we did not observe the significant changes on these makers between active and remission phase [14]. Several speculations were considered. First, the timing of blood sampling during flares may miss the peak levels of C5a and C5b-9 in circulation due to their short half-lives. Because we collected blood from all patients immediately at presentation and measured sequentially, this might be excluded. Second, these terminal complement products generated and deposited at the sites of damaged tissue during complement activation. Specifically, this immune complex-induced local complement activation may not be faithfully reflected by the concentration of circulating active complement products. Our immunofluorescence stains revealed the glomerular C5b-9 depositions on renal tissues from patients with lupus nephritis but not from disease-controls, and normal subjects. In accordance with our finding, Sato et al. and Song et al. showed the strong glomerular C5b-9 deposition and suggested that complement-mediated tissue injury might play an important role of lupus nephritis [14, 16]. These findings indicated that C5b-9 depositions on tissue targeted might exactly reflect the real complement activation, which leads to the development of tissue injury.

As mentioned above, serum CFI is likely to have roles in complement activation. Further potential therapies may focus on the termination of complement activation by either the supplementation of soluble CFI by plasma infusion or blockade of the downstream products of C3 activation. A recent study has demonstrated that treatment with soluble CRPs in pregnant mice treated with human IgG containing antiphospholipid antibodies prevents fetal loss by inhibiting complement activation [17]. The administration of an anti-C5 monoclonal antibody has been proven to be effective in patients with complement dysregulation hemolytic uremic syndrome and NZB/WF1 mouse model by blocking the development of C5b-9, which led to the retardation of glomerular damage and an increase in survival, respectively [18, 19].

The current study has some limitations. Due to the strict criteria, four patients who did not reach to remission were excluded. We recognized that the presented sequels might have been underestimated. Actually, the patients we enrolled were well-characterized pediatric-onset lupus with consistent gender distribution, typical clinical features, and bona fide flares by the significant differences in SLEDAI, serum C3 and C4, anti-dsDNA, and hematologic features in active phase [20–26]. The second limitation is the possible impacts of different treatments on the serum CRPs levels. Actually, we treated our cohort patients with corticosteroids, or methylprednisolone pulse therapy, or/and cyclophosphamide or mycophenolate mofetil according to their disease activity and the presented consequences. These may decrease this potential impact. The third limitation is that we measured circulating CD46 rather than membrane CD46 due to unavailable fresh blood samples by the nature of retrospective study. However, the circulating CD46 levels have been suggested to be a marker to reflect *in vivo* activation of complement system by Kawano et al. [12].

In conclusion, we demonstrated serum CFI levels not only correlated with the disease activity, but also associated the serum C3 level and hematological features of SLE. These findings indicate that serum CFI is a potential new serum biomarker.

## MATERIALS AND METHODS

The study protocol was approved by the Ethics Committee on Human Studies at Chang Gung Memorial Hospital, in Taiwan, R.O.C. (IRB 102-5544A3). The subjects were given a detailed description of the study before they provided informed consent.

### Subjects

One hundred and forty patients from our pediatric SLE cohort study were selected for study. All these patients fulfilled the American College of Rheumatology revised criteria and received clinical and laboratory evaluations at least every 2 months [27, 28]. We included one hundred and two pediatric-onset SLE patents that had flares followed by reaching into remission during the 2006-2015 period. Patients who (1) did not reach to remission phase; (2) lack of sufficient blood samples for analysis; (3) had less than 1 year follow-up; (4) loss of follow-up were excluded. In addition, we enrolled patients with their first flare only if several episodes occurred during study period. Forty patients were selected to study finally (Figure 1).

### Clinical features

The phenotypes included clinical presentation, laboratory data, treatment and clinical outcome in the active and remission phases of SLE. The active and remission phases of SLE were defined as disease activity of SLE (SLEDAI) scores > 10 and < 4 for 2 consecutive visits, respectively [29]. The renal SLEDAI score (range 0–16) represents the sum of the renal items of the SLEDAI-2K. If present, each of the four SLEDAI-R items receives a score of 4: proteinuria of > 0.5 gram/day, hematuria and pyuria (both > 5 cells/high power field), and cellular casts [30]. Clinical presentations, including fever, rash, oral ulcer, non-erosive arthritis, photosensitivity, hematologic disorders (anemia, thrombocytopenia, and lymphopenia), renal disorders (proteinuria and hematuria), and neuropsychiatric disorders (seizure and psychosis) were recorded. All enrolled patients were treated with either intravenous methylprednisolone pulse therapy or high-dose oral prednisolone (2 mg/kg/day) for their flares, and intravenous cyclophosphamide or mycophenolate mefetil for those with class III, IV and V nephritis. Oral mycophenolate mefetil was given for patients with nephritis refractory or intolerant to cyclophosphamide. Histological classifications of lupus nephritis were determined according to the 2003 ISN/RPS system [31].

### Biochemical characteristics

Biochemical data, including serum creatinine, blood urea nitrogen, serum C3, serum C4, serum anti-dsDNA, white blood count, platelets (PLT), hemoglobin (Hgb), and urinalysis were analyzed on the days of determination of active and remission phases.

### Serum complement regulatory proteins and terminal complement products

Blood samples obtained on the days of active phase before medication and remission phase were studied for complement regulatory proteins and terminal complement products in the same flare-up. The levels of serum CFH (Abnova), CFI (LSBio), C5a (BD OptEIA™ Human C5a), and C5b-9 (Blue Gene Biotech) were measured using ELISA according to the manufacturer’s guidelines. The linear portion of the standard curve was subsequently used for the measurement of serum CFH, CFI, C5a, and C5b-9. All assays were run in duplicate, and when standard errors were over 10%, samples were routinely re-analyzed. The method of detecting serum CD46 by sandwich ELISA was the same as previously reported [12]. The quantities of serum CD46 in the samples were determined based on the A_450nm_ of the purified CD46.

### Immunofluorescence stain of renal tissue

For evaluating the possible complement activation on local tissue during flares, we performed immunofluorescence stain for the expression of active complement products on glomeruli obtained from patients with biopsy-proven nephritis in active phase, normal subjects, and disease controls. Real tissues selected as normal and disease controls were from subjects with incidental trauma and focal segmental glomerulosclerosis, respectively. The quantities of C5b-9 were analyzed at 400x magnification. The numerical value for overall intensity, intensity score, is based on a 5-point system: 0, 1, 2, 3, and 4 (for none, faint, light, medium, and high staining).

### Statistical analysis

Variables are represented as the mean ± SD. Student’s *t*-test was performed for the comparisons of continuous parametric variables between two groups. Statistical analyses were performed using SPSS Software (version 16.0; SPSS Inc., Chicago, IL, USA). *P* values < 0.05 were considered statistically significant.

## Abbreviations

SLE: Systemic Lupus Erythematosus
SLEDAI: Systemic Lupus Erythematosus Disease Activity Index
CRPs: Complement Regulatory Proteins
CFI: Complement Factor I
CFH: Complement Factor H
CD46: Cluster of Differentiation 46
